# Sensor-Based Ozone Monitoring and Forecasting in a Synchrotron Radiation Laboratory Using Autoregressive Integrated Moving Average Models

**DOI:** 10.3390/s26072251

**Published:** 2026-04-06

**Authors:** Po-Jiun Wen, Kuo-Wei Wu, Liang-Chen Ho, Chieh-Han Yang, Tsung-Hung Tsai, Shih-Hau Fang

**Affiliations:** 1 Radiation and Operation Safety Division, National Synchrotron Radiation Research Center, Hsinchu 300092, Taiwan; wen.pj@nsrrc.org.tw; 2 Electrical Engineering Department, Yuan Ze University, Taoyuan 32003, Taiwan; wux369865@gmail.com (K.-W.W.); ricky911126@gmail.com (L.-C.H.); poteddaniel0528@gmail.com (T.-H.T.); 3 Inventec Corporation, Taoyuan 333547, Taiwan; yoyo5757jack@gmail.com; 4 Electrical Engineering Department, National Taiwan Normal University, Taipei 106308, Taiwan

**Keywords:** ozone sensing, environmental monitoring, sensor data analysis, ARIMA forecasting, laboratory safety

## Abstract

**Highlights:**

**What are the main findings?**

- First on-site machine learning implementation for ozone prediction at the National Synchrotron Radiation Research Center (NSRRC), based on controlled real experimental data.
- The optimized ARIMA model outperformed LSTM and Linear Regression in small-sample forecasting, achieving R^2^ up to 89.5% and MAPE below 1%.

**What are the implications of the main findings?**

- Provides the first data-driven predictive framework for ozone safety management in synchrotron laboratory environments.
- Demonstrates that lightweight and interpretable time-series models are more reliable than deep learning in precision facilities with limited sensing data.

**Abstract:**

Ozone monitoring in laboratory environments is essential for ensuring personnel safety and maintaining stable experimental conditions, particularly in enclosed facilities where ozone may accumulate during high-energy radiation operations. This study investigates the short-term prediction of ozone concentration using data obtained from a sensor-based ozone monitoring system deployed at the National Synchrotron Radiation Research Center (NSRRC). Ozone concentration measurements were collected using a UV absorption-based ozone analyzer and analyzed as a time-series dataset under controlled experimental conditions. Three forecasting models—Autoregressive Integrated Moving Average (ARIMA), Long Short-Term Memory (LSTM), and linear regression—were evaluated for short-term ozone concentration prediction. Experimental results indicate that the ARIMA model provides superior predictive performance for the small-sample dataset used in this study. In the Right direction, ARIMA achieved R^2^ values of 89.5%, 86.3%, and 81.1% at distances of 5 cm, 10 cm, and 15 cm, respectively, while also demonstrating stable performance in the Up direction. The results highlight the effectiveness of classical time-series models for sensor data analysis in environments with limited sensing data. The proposed framework demonstrates the potential of integrating sensing devices with predictive data analytics to support real-time environmental monitoring and safety management in laboratory facilities.

## 1. Introduction

Environmental monitoring systems increasingly rely on sensor technologies integrated with intelligent data analysis methods to detect and predict hazardous gas concentrations in industrial and laboratory environments. Among these gases, ozone (O_3_) is a strong oxidizing agent that poses significant respiratory health risks when it accumulates in enclosed spaces [1,2]. Recent advances in environmental sensing have emphasized the integration of sensor measurements with predictive data-driven models. By analyzing time-series data collected from sensing devices, predictive algorithms can estimate future pollutant concentrations and provide early warnings before hazardous thresholds are reached. Such approaches extend traditional sensing systems into predictive monitoring platforms, enhancing environmental safety management and decision support in controlled laboratory environments. The proposed framework aims not only to analyze ozone concentration dynamics but also to support the development of predictive sensor-based monitoring systems for laboratory safety management.

In facilities that utilize high-energy radiation sources, such as synchrotron laboratories, ozone can be generated through the interaction of ultraviolet radiation with atmospheric oxygen. Continuous monitoring using gas sensing instruments is therefore essential to ensure laboratory safety and maintain stable operating conditions. At the National Synchrotron Radiation Research Center (NSRRC), synchrotron light sources generate ozone during beamline operations. Because ventilation systems are sometimes temporarily deactivated to maintain experimental stability, ozone concentration can increase rapidly inside the Hutch area, potentially exposing personnel to hazardous levels [3,4,5,6]. While previous research has explored gas concentration forecasting in industrial or mining environments [7,8,9,10,11], studies targeting ozone dynamics in synchrotron-based laboratory settings remain scarce. Under these circumstances, sensor-based ozone monitoring systems are critical for detecting concentration changes and enabling timely safety responses.

Against this backdrop, this study represents the first known application of machine learning to forecast ozone concentrations within the NSRRC. Data collection and model training were conducted entirely on-site in a controlled, enclosed experimental setting, reflecting real operational conditions. To improve model stability and accuracy, the dataset was carefully filtered based on spatial distribution characteristics: in the vertical direction, data from the lower region were excluded due to extremely low and unstable concentrations; in the horizontal direction, the right side was selected as the representative sampling location. Ultimately, one sampling point from the right side and one from the upper region were chosen to represent meaningful variations in ozone dissipation behavior.

To meet the needs of real-time, resource-efficient, and accurate forecasting, this study employs the Autoregressive Integrated Moving Average (ARIMA) model and compares its performance against Long Short-Term Memory (LSTM) networks and linear regression models. The ARIMA model is well-suited for short-term forecasting with small, non-seasonal datasets and has been widely used in communication delay prediction, environmental and industrial time series prediction tasks [12,13,14]. Its core components—Autoregression (AR), Integration (I), and Moving Average (MA)—allow it to effectively capture trends, autocorrelation, and stabilize non-stationary data.

Therefore, this study aimed to employ the Autoregressive Integrated Moving Average (ARIMA) model to forecast indoor ozone concentration trends at the National Synchrotron Radiation Research Center (NSRRC), marking the first application of machine learning within this unique research facility. The results are expected to provide valuable insights for laboratory personnel, facility managers, and safety engineers, supporting timely risk mitigation and the enhancement of air quality control strategies. This study not only addresses a previously unexplored issue at NSRRC but also offers practical guidance for environmental safety planning and management in other advanced laboratory environments.

## 2. Related Work

Time series forecasting has become an essential tool in various fields, ranging from finance and economics [15], environmental science [16], and supply chain management [17]. Over the past few decades, various models have been developed to address the complexities inherent in time series data. Among these models, traditional statistical approaches like the ARIMA model and modern deep learning architectures such as LSTM networks have gained significant attention.

ARIMA, introduced by Box and Jenkins in the 1970s [18], is renowned for its ability to handle noisy data and make accurate predictions by combining autoregressive, differencing, and moving average components [19]. It has been applied in diverse areas such as predicting crime rates [20], user data growth [21], stock prices [22], and disease outbreaks [23], where semantic information was integrated with ARIMA to improve forecasting accuracy for dengue case trends. Researchers use criteria like the Akaike Information Criterion (AIC) and Bayesian Information Criterion (BIC) to select optimal model parameters, balancing complexity and accuracy [24].

The integration of ARIMA with advanced models like self-paced ARIMA [25], LSTM, and hybrid neuro-fuzzy systems [26,27,28] has further improved prediction accuracy and robustness, particularly in gas concentration forecasting tasks. Li and Hu [28] proposed a novel ARIMA-based neuro-fuzzy approach combined with swarm intelligence, demonstrating superior performance in complex time series environments. Additionally, model selection techniques involving AIC, BIC, and adjusted R^2^ provide a solid foundation for determining the best model fit. For gas concentration prediction, Huang et al. [8] integrated ARIMA with the Spark Streaming framework and Support Vector Machines (SVM) to enhance real-time monitoring capabilities in coal mines.

Recent studies have further explored hybrid frameworks that combine ARIMA with advanced deep learning architectures. Hybrid ARIMA–LSTM models have been successfully applied in transformer oil temperature prediction, vehicle speed forecasting, and reservoir inflow estimation [29,30,31]. These models leverage ARIMA’s ability to capture linear structures while utilizing LSTM to model nonlinear and long-term dependencies. Furthermore, more sophisticated architectures incorporating BiLSTM, optimization algorithms, and ensemble learning techniques have been proposed for financial forecasting, achieving enhanced robustness and accuracy in highly dynamic systems [32].

LSTM, a deep learning model, addresses the vanishing and exploding gradient problems in traditional recurrent neural networks by using a memory cell structure with forget, input, and output gates [33,34]. This structure allows LSTM to effectively capture long-term dependencies, making it suitable for applications in financial markets [35,36], traffic prediction [37], intrusion detection [38], and flood prediction [39]. For gas concentration prediction, Zhang et al. [40] used a PSO-LSTM model to predict the concentration of ethylene and methane in air, improving the prediction accuracy of mixed gas concentrations. Peng et al. [41] used a combined model based on indicators, dynamic optimization, and Bi-directional Long Short-term Memory (Bi-LSTMs) to improve the safety production efficiency of coal mines. Similarly, Faruk [42] presented a hybrid model combining ARIMA and neural networks for water quality prediction, highlighting the complementary strengths of statistical and machine learning techniques in environmental time series forecasting. Both models offer robust predictive capabilities, with ARIMA excelling in handling stationary data and LSTM capturing complex, non-linear dependencies.

Linear regression, one of the most fundamental and widely used statistical models, has long served as a baseline method in time series forecasting and predictive analytics. Its simplicity, interpretability, and efficiency make it particularly suitable for scenarios where the relationships between variables are assumed to be linear and where data exhibit minimal complexity or noise [43]. In environmental modeling, linear regression has been applied to estimate pollutant concentrations, analyze temporal trends, and evaluate the impact of external factors such as weather conditions or industrial activities on air quality [44]. For example, Gao et al. [45] utilized multivariate linear regression to examine the relationship between ozone levels and meteorological parameters, achieving reasonably accurate short-term forecasts.

Despite its strengths in computational efficiency and transparency, linear regression models often fall short when dealing with complex, nonlinear patterns commonly present in gas concentration or environmental data. To address these limitations, researchers have proposed hybrid approaches, integrating linear regression with more advanced models such as LSTM or Support Vector Machines (SVM), enhancing both predictive performance and model robustness [46,47]. These hybrid methods exploit the linear model’s interpretability while leveraging the nonlinear capabilities of machine learning techniques, thereby improving accuracy in real-world forecasting tasks.

## 3. Methodology

**Figure 1:**
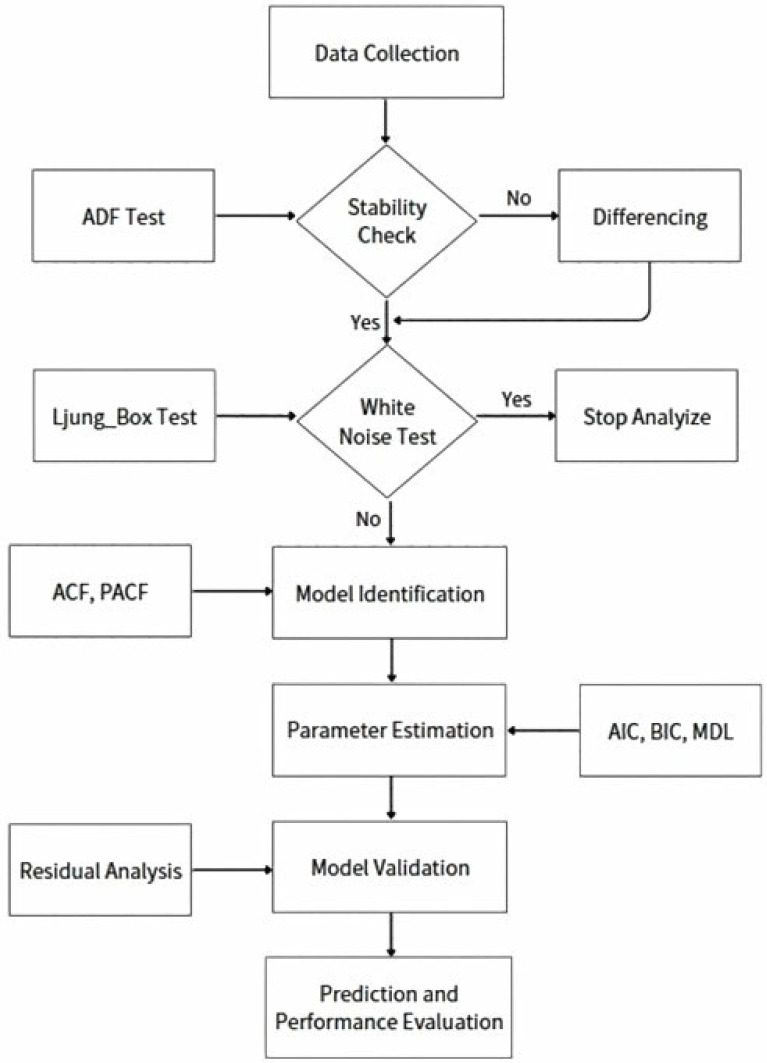
This research process framework.

This study’s framework aims to predict ozone concentration time series changes using the ARIMA model to assess environmental safety conditions. The research framework is illustrated in Figure 1, which serves as the main research process flowchart. The entire research process involves several key steps. First, data segments are manually selected and reviewed from relevant sources, ensuring data quality and applicability by avoiding segments with abrupt decay in the initial stages. The stability of the time series is then assessed using the Augmented Dickey–Fuller (ADF) test [48], where a *p*-value less than 0.01 indicates data stability; otherwise, differencing is performed to achieve stability, determining the order of differencing. The model’s residuals are checked using the white noise test [49] to ensure they exhibit randomness with stable fluctuations and low autocorrelation, indicating a good fit. The Ljung–Box test [50] is also conducted to detect autocorrelation, ensuring the residuals are independently distributed.

If the *p*-value is significantly greater than 0.05, the model is considered to have a good fit; otherwise, further optimization is required. The autocorrelation function (ACF) [48] is used to determine the number of autoregressive (AR) terms, while the Partial Autocorrelation Function (PACF) [51] helps identify the number of moving average (MA) terms in the ARIMA model. Model parameters are estimated using information criteria such as Akaike Information Criterion (AIC) and Bayesian Information Criterion (BIC) [52], along with the Minimum Description Length (MDL) method [53]. The model with the lowest AIC, BIC, or MDL value is selected to achieve the best fit.

The MDL method is introduced as an auxiliary tool for parameter estimation, which automatically selects the model complexity based on the structure of the data, balancing fitting error and model complexity. Compared to AIC and BIC, MDL emphasizes model simplicity, preventing overfitting, thereby improving the model’s predictive and generalization capabilities. These estimation methods help ensure that the chosen model maintains stable and accurate forecasting ability across different data variations.

A Q–Q plot is used to verify whether the residuals follow a normal distribution, ensuring that they are white noise. The Durbin–Watson statistic and other related functions are used to examine autocorrelation in the residuals. The predictive performance of the ARIMA model is evaluated using metrics such as R-squared (R^2^), Mean Absolute Percentage Error (MAPE), and Root Mean Squared Error (RMSE), to establish an accurate predictive model for evaluating the temporal trends in ozone concentration.

In the data collection and processing phase, the research focuses on ensuring the quality and applicability of the data to effectively support ARIMA model development and prediction analysis. Initially, suitable data segments are manually selected from the collected dataset, with particular attention to excluding segments with abrupt declines in ozone concentration that could cause prediction errors. The selected data undergoes further analysis to understand trends, characteristics, and variations. Statistical properties such as mean and standard deviation are evaluated to ensure data representativeness and consistency. Irregular data changes are processed using smoothing techniques like moving averages to minimize noise and anomalies in the prediction model.

The stability of the data series is crucial for establishing an effective ARIMA model. The ADF test checks the stability of the time series, with a *p*-value less than 0.01 indicating stationarity. Non-stationary series undergo differencing to achieve stationarity, where the differencing order is a key parameter in ARIMA, often determined by the need to eliminate trends or seasonal components. After differencing, interpolation may be used to supplement the data if necessary, ensuring sufficient data for model training.

The parameter estimation phase involves determining the autoregressive (AR) and moving average (MA) components of the model. The ACF plot identifies autocorrelation in the time series, helping determine the number of moving average terms, while the PACF plot identifies partial autocorrelation, helping determine the autoregressive terms. These plots provide initial estimates of the ARIMA model parameters and offer insights into potential seasonal and non-seasonal patterns in the data. Once the parameters are determined, the AR and MA components are integrated to form the complete ARIMA model, which considers both past values and past forecast errors in its predictions [54].

This methodology requires a deep understanding of time series analysis and proficiency with statistical tools to interpret ACF and PACF plots correctly, ensuring accurate parameter estimation. By following these steps, the ARIMA model can predict future data points effectively, enhancing prediction accuracy and reliability.

Akaike Information Criterion (AIC) and Bayesian Information Criterion (BIC) are widely used model selection criteria based on information theory to evaluate the goodness of different ARIMA model parameter combinations (p, d, q) and choose the most suitable model. Both AIC and BIC aim to quantify information loss but differ slightly in their calculations. AIC is defined as per Equation (1)

(1) $$AIC=2k-2\ln\left(L\right)$$

where k is the number of model parameters and L is the maximum likelihood estimation of the model. AIC encourages a good data fit but penalizes excessive model parameters. BIC, on the other hand, is defined as per Equation (2)

(2) $$BIC=\ln\left(n\right)k-2\ln\left(L\right)$$

where n is the sample size, k is the number of model parameters, and L is the maximum likelihood estimation of the model. BIC imposes a heavier penalty for the number of parameters, favoring simpler models with larger sample sizes.

In addition to AIC and BIC, this study also introduces the Minimum Description Length (MDL) criterion as a supplementary model selection method. MDL aims to minimize the length of the description of the data, considering not only the model fit but also emphasizing model simplicity. The MDL formula is given as

(3) $$MDL=n*\ln\left(\sigma^{2}\right)+k*ln(n)$$

where n is the sample size, $\sigma^{2}$ is the error variance of the model, and k is the number of model parameters. The main feature of MDL is that it penalizes the complexity of the model (i.e., the number of parameters), placing more emphasis on controlling the error during the penalization process, thus preventing overfitting. Compared to AIC and BIC, MDL tends to select solutions that effectively fit the data without excessively increasing model complexity.

To apply AIC, BIC, and MDL for model selection, the AIC, BIC, and MDL values for each (p, d, q) combination are calculated, and the combination with the lowest AIC, BIC, or MDL value is chosen based on the principle of minimizing information loss, seeking a model that balances fit and complexity. Once the model with the lowest AIC, BIC, or MDL value is selected, further evaluation and validation are necessary, including checking residuals to ensure they meet the white noise characteristics and performing cross-validation or testing on independent datasets. These steps ensure that the model not only performs well on the training set but also demonstrates good predictive ability, providing a robust framework to guarantee the accuracy of forecasting performance.

## 4. Research Results

**Figure 2:**
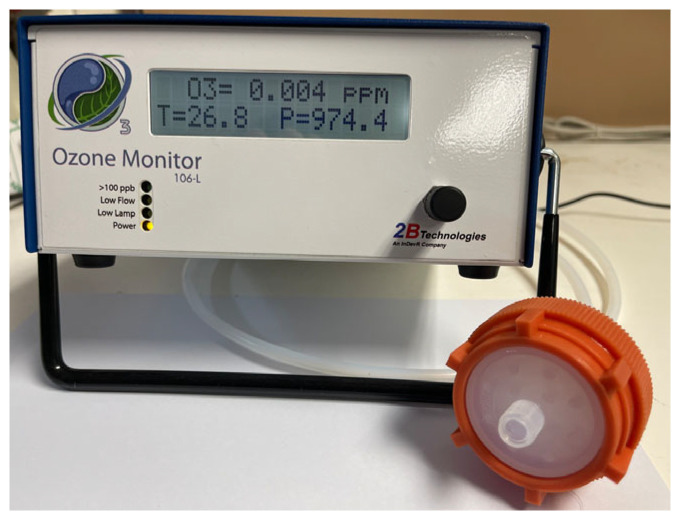
Appearance of measuring instrument 106-L.

This study utilized real data from the National Synchrotron Radiation Research Center (NSRRC, Hsinchu, Taiwan) and was conducted under conditions in the Hutch with a storage ring energy of 3.0 GeV and a current of 500 mA. The light source was turned on while the ventilation and air conditioning inside the beamline cabin were turned off, with the humidity remaining constant. The focus was on monitoring changes in ozone concentration from 300 to 850 s after the light source was turned off. Measurements were performed using a 106-L instrument. As shown in Figure 2, the ozone concentration was measured using a Model 106-L Ozone Monitor (2B Technologies, Boulder, CO, USA), which operates based on UV absorption at 254 nm. The main specifications of the instrument are summarized below as Table 1. The Model 106-L ozone monitor provides a linear measurement range of 0–100 ppm with a resolution of 0.01 ppm. The precision (1σ for a 10 s average) is specified as the greater of 0.01 ppm or 2% of the reading, while the limit of detection (2σ for a 10 s average) is 0.02 ppm. The instrument accuracy is also given as the greater of 0.01 ppm or 2% of the measured value. In terms of stability, the baseline drift is less than 0.01 ppm per day and less than 0.03 ppm per year, and the sensitivity drift is less than 1% per day and less than 3% per year. The ozone measurements are obtained with a 2 s measurement interval (0.5 Hz), and the instrument allows multiple data averaging options including 10 s, 1 min, 5 min, and 1 h. The monitor includes temperature and pressure correction, and a DewLine™ humidity control system to reduce the influence of moisture on measurements. The required sample flow rate is 0.3–1.5 L/min (nominal 1 L/min). Regarding calibration, the instrument is NIST-traceable, and annual calibration is recommended by the manufacturer to ensure long-term measurement accuracy and stability. It has been certified by the United States Environmental Protection Agency (EPA) as a Federal Equivalent Method (FEM) instrument, ensuring compliance with the U.S. Clean Air Act (certification: EQOA-0914-218). These features make it an ideal choice for precise ozone monitoring in various environmental and industrial settings.

**Figure 3:**
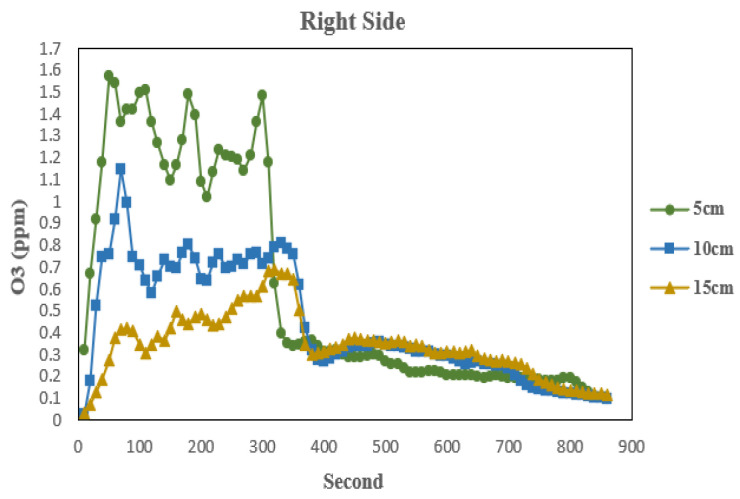
Ozone measurement data at different distances.

The experimental measurement method was as follows: In one Hutch, the light source was turned on while all exhaust fans were turned off. After measuring the ozone production within the Hutch for 300 s, the light source was turned off and the door was opened. Using the natural diffusion of ozone, the local concentration at the measurement point was continuously monitored until it decreased to 0.1 ppm (Time Weighted Average, TWA, this concentration is the permissible threshold), with a total collection time of approximately 850 s. During this measurement period, the instrument’s component temperature increased by 0.1–0.6 °C, the component pressure varied from −2 to 1.4 torr, the component pumping flow rate changed from −104 to 75 cc/min, and the photodiode voltage increased by 0.0053–0.3374 V. All these instrument parameter changes remained within normal operating ranges and did not affect the overall experiment. Ozone measurement data obtained at different distances along the vertical plane to the right of the light source are shown in Figure 3.

According to the instrument specifications, the analyzer performs measurements with a sampling interval of 2 s, corresponding to a sampling frequency of 0.5 Hz. Therefore, ozone concentration data were recorded every 2 s throughout the experiment. This high temporal resolution, with the average of the sampled data uploaded every 10 s, allows for the capture of rapid ozone concentration changes during both the ozone formation and subsequent diffusion phases. The ozone analyzer used in this study performs measurements at a 2 s interval, which results in approximately 425 data samples per experiment (850 s/2 s). These measurements constitute the time-series dataset used for training and testing the prediction models. This study will focus on accurately predicting the time required for the ozone concentration to drop to 0.1 ppm after the light source in the Hutch is turned off. Since the ozone concentration data constitutes a time series, a time series forecasting model can be used to predict its concentration over time and determine when the ozone concentration reaches 0.1 ppm.

**Figure 4:**
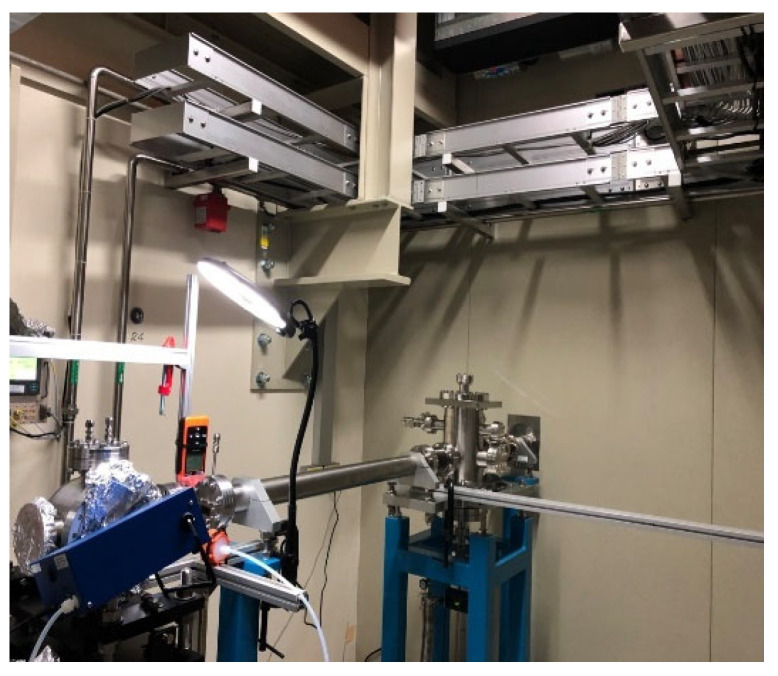
Laboratory light source ozone measurement environment.

The National Synchrotron Radiation Research Center conducted measurements at different distances and positions from the light source to better understand the ozone distribution. The measurement conditions came from six different settings, as shown in Table 2. The experimental environment is detailed in Figure 4.

In the model training and testing process, the data was divided into two segments: the data from 300 to 660 s (180 samples) was used as the training set, while the data from 660 to 700 s (20 samples) served as the test set. This study was implemented using Python (version 3.x), where the ARIMA model was developed using the statsmodels library (version 0.14.6). The LSTM and Linear Regression models were implemented using TensorFlow (version 2.20.0) with the Keras API (version 3.10.0). After training the models, predictions were made using the data from 660 to 700 s, and the predicted results were compared with the actual ozone concentration data during that period.

To assess the predictive performance of the models, the Root Mean Square Error (RMSE), Coefficient of Determination (R^2^), and Mean Absolute Percentage Error (MAPE) were calculated. These metrics help identify which model performs best in this application scenario. Each data record includes the time (field name: time) and ozone concentration (field name: ppm). By using these evaluation metrics, the predictive performance of the ARIMA, LSTM, and Linear Regression models was objectively compared to determine the most suitable model for forecasting ozone concentration changes.

Table 3 presents the R^2^, RMSE, and MAPE performance indicators obtained from the ARIMA, LSTM, and Linear Regression models applied to the right-side dataset. The results demonstrate that the ARIMA model outperforms both the LSTM and Linear Regression models across all three distance measures, particularly for the 5 cm data on the right side. In this case, ARIMA achieved an impressive R^2^ of 89.5%, an RMSE of 0.001, and a MAPE of 0.41%. The LSTM model also showed commendable performance with the 5 cm data, but as the distance increased, the results gradually declined, with the R^2^ for the 15 cm data falling below zero. The Linear Regression model also performed well with the 10 cm data on the right side, achieving an R^2^ of 66.4%, but for more complex data, the simpler nature of Linear Regression failed to fully capture the underlying patterns.

**Figure 5:**
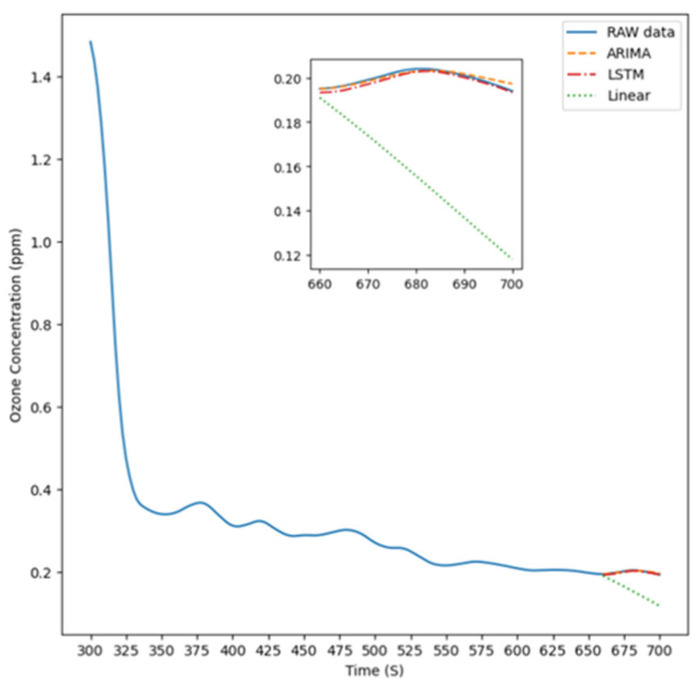
The prediction result is 5 cm to the right.

Figure 5 illustrates the experimental setup for Experiment Condition 1, where the instrument was positioned 5 cm to the right of the light source to measure ozone concentration. The figure features four distinct lines representing the prediction results of the ARIMA model, LSTM model, linear model, respectively, compared with the raw data, which are used to highlight the actual differences among these approaches.

**Figure 6:**
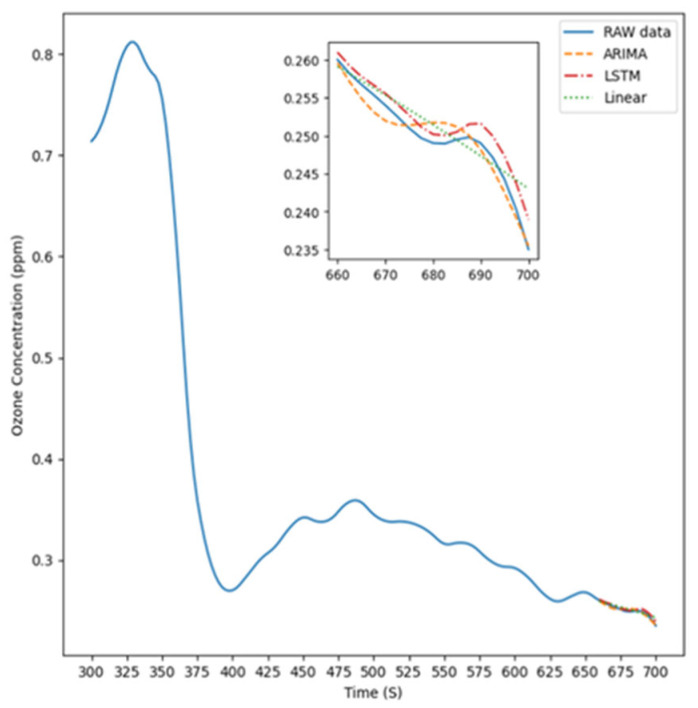
The predicted result is 10 cm to the right.

Figure 6 illustrates the experimental setup for Experiment Condition 2, where the instrument was positioned 10 cm to the right of the light source to measure ozone concentration. The figure features four distinct lines representing the prediction results of the ARIMA model, LSTM model, linear model, and the original data, respectively, compared with the raw data, which are used to highlight the actual differences among these approaches.

**Figure 7:**
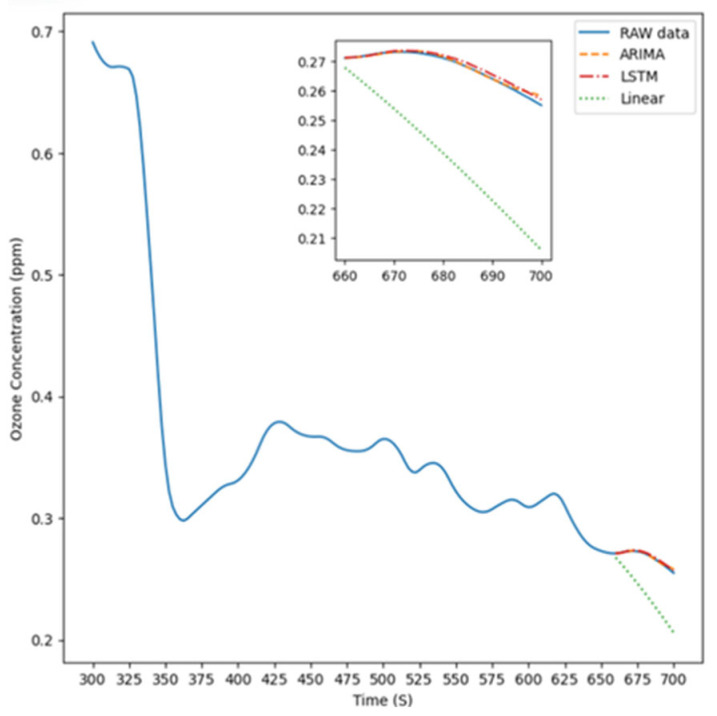
The predicted result is 15 cm to the right.

Figure 7 illustrates the experimental setup for Experiment Condition 3, where the instrument was positioned 15 cm to the right of the light source to measure ozone concentration. The figure features four distinct lines representing the prediction results of the ARIMA model, LSTM model, linear model, and the original data, respectively, compared with the raw data, which are used to highlight the actual differences among these approaches.

Table 4 presents the performance indicators, including R^2^, RMSE, and MAPE, obtained from the ARIMA, LSTM, and Linear models applied to the Up-direction dataset. The results indicate that the ARIMA model consistently outperformed both the LSTM and Linear models across all three distances, with particularly strong performance observed at 30 cm. At this distance, the ARIMA model achieved an R^2^ of 79.73%, an RMSE of 0.0021, and a MAPE of 0.68%, demonstrating its high accuracy. The LSTM model, while showing some positive results, struggled as the distance increased, with its R^2^ falling to negative values at 10 cm and 30 cm. The Linear model exhibited significant underperformance, particularly at all distances, with R^2^ values that were negative, highlighting its inability to model the data effectively in this context. This suggests that ARIMA was the most reliable model for predicting ozone concentrations in the Up-direction dataset, particularly for more complex and variable data at larger distances.

**Figure 8:**
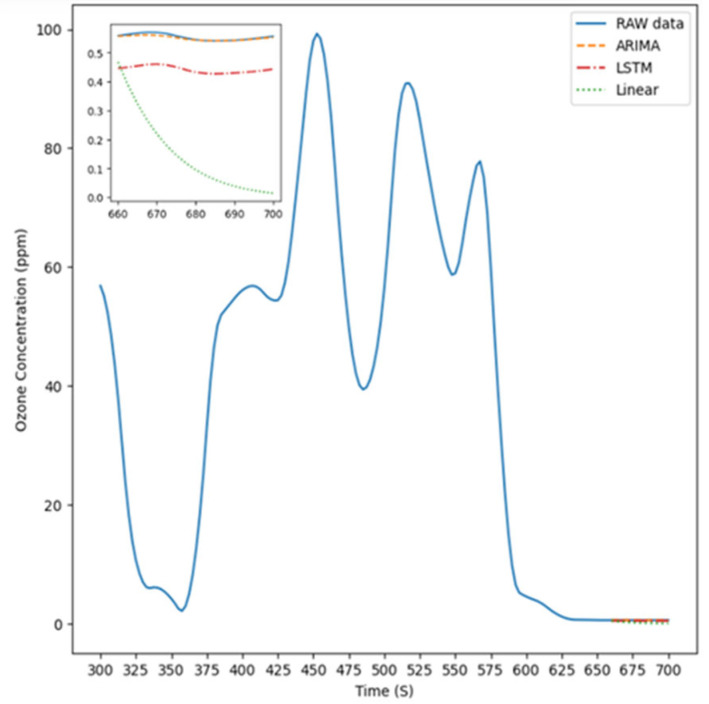
The predicted value is 5 cm up.

Figure 8 illustrates the experimental setup for Experiment Condition 4, where the instrument was positioned 5 cm on the up of the light source to measure ozone concentration. The figure features four distinct lines representing the prediction results of the ARIMA model, LSTM model, linear model, and the original data, respectively, compared with the raw data, which are used to highlight the actual differences among these approaches.

**Figure 9:**
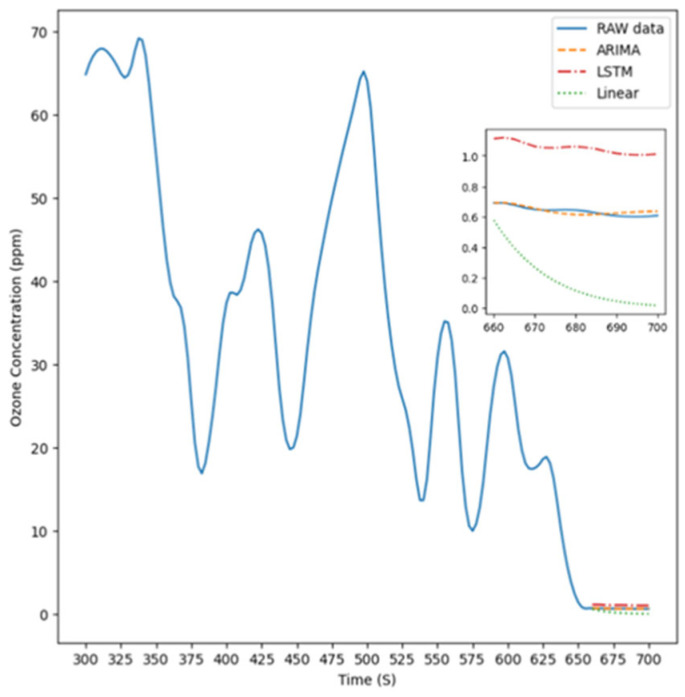
The predicted value is 10 cm up.

Figure 9 illustrates the experimental setup for Experiment Condition 5, where the instrument was positioned 10 cm on the up of the light source to measure ozone concentration. The figure features four distinct lines representing the prediction results of the ARIMA model, LSTM model, linear model, and the original data, respectively, compared with the raw data, which are used to highlight the actual differences among these approaches.

**Figure 10:**
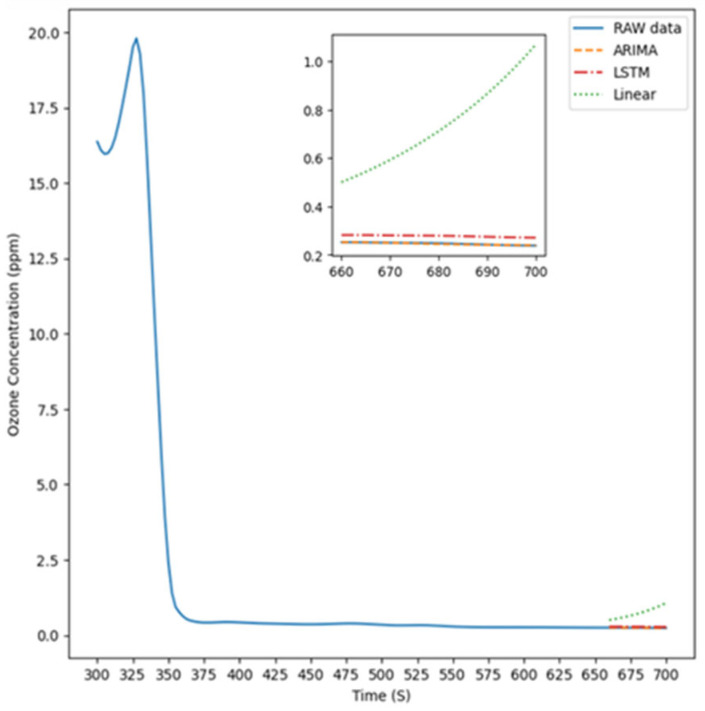
The predicted value is 30 cm up.

Figure 10 illustrates the experimental setup for Experiment Condition 6, where the instrument was positioned 30 cm on the up of the light source to measure ozone concentration. The figure features four distinct lines representing the prediction results of the ARIMA model, LSTM model, linear model, and the original data, respectively, compared with the raw data, which are used to highlight the actual differences among these approaches.

**Figure 11:**
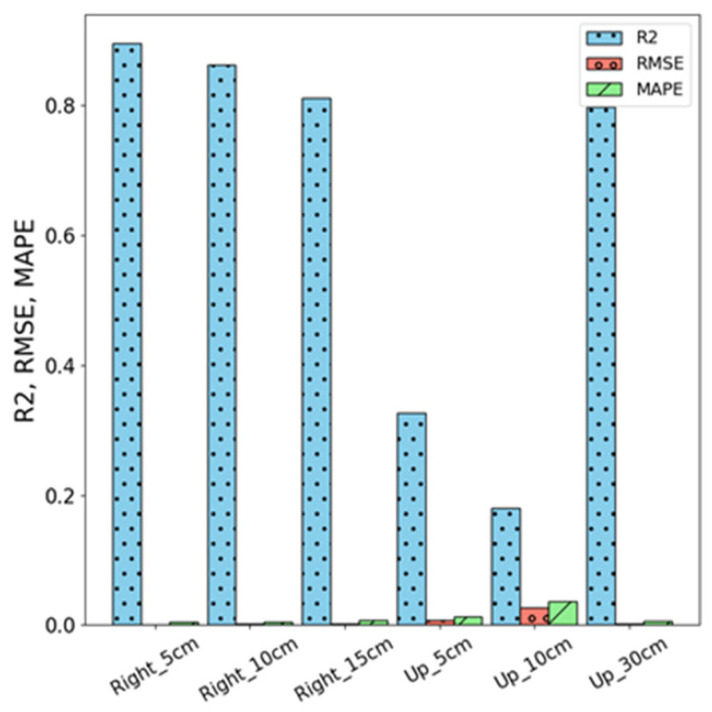
Performance of ARIMA models under different conditions.

Figure 11 shows the comparison of the three evaluation metrics, R^2^, RMSE, and MAPE performance of ARIMA, demonstrating its superior explanatory power, prediction accuracy, and stability. At Right-side measurement positions (5 cm, 10 cm, and 15 cm), ARIMA achieves R^2^ values of 89.5%, 86.3%, and 81.1%, respectively, showcasing strong explanatory power. Even at the Up-direction (5 cm, 10 cm, and 30 cm), where performance slightly declines, ARIMA maintains higher R^2^ values of 32.7%, 18.0%, and 79.7% compared to its counterpart. Similarly, ARIMA exhibits minimal RMSE values, such as 0.001, 0.002, and 0.002 at Right-side positions, and 0.008, 0.027, and 0.002 at the Up-direction, reflecting its precise predictions. Moreover, ARIMA consistently records low MAPE values across all positions, with examples like 0.41%, 0.55%, and 0.79% at Right-side positions, indicating remarkable stability and accuracy.

**Figure 12:**
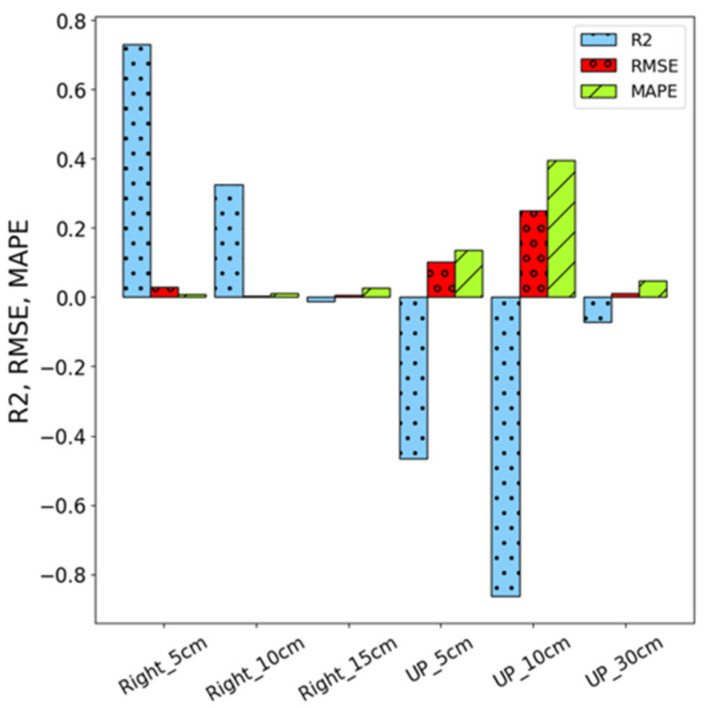
Performance of LSTM models under different conditions.

Figure 12 shows the comparison of the three evaluation metrics, R^2^, RMSE, and MAPE performance of the LSTM model, which exhibits significantly weaker performance across all metrics, with better results at Right-side positions compared to the Up-direction. At Right-side positions (5 cm, 10 cm, and 15 cm), LSTM achieves R^2^ values of 73.2%, 35.5%, and −1.2%, respectively, whereas at the Up-direction (5 cm, 10 cm, and 30 cm), the R^2^ values drop sharply to −46.5%, −86.3%, and −7.1%. Similarly, the RMSE values reveal smaller errors at the Right-side positions, with 0.030, 0.004, and 0.007, but these errors increase significantly at the Up-direction, reaching 0.103, 0.251, and 0.012. The MAPE values also follow this pattern, with 1.0%, 1.3%, and 2.7% at the Right-side positions, which are notably better than the larger errors observed at the Up-direction.

**Figure 13:**
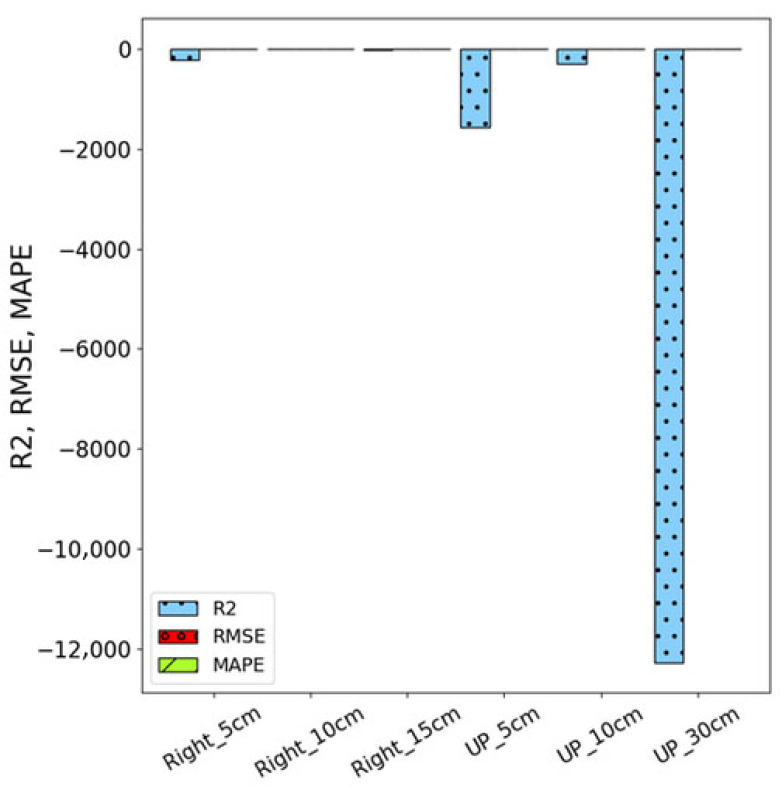
Performance of linear models under different conditions.

Figure 13 shows the comparison of the three evaluation metrics, R^2^, RMSE, and MAPE performance of the Linear model. The Linear model performs poorly in both Right-side and Up-direction measurements across all evaluation metrics. In the Right-side direction, the model shows negative R^2^ values at 5 cm (−219.8%) and 15 cm (−25.4%), indicating a failure to capture data patterns, while at 10 cm, it achieves a modest R^2^ of 66.4%. The RMSE values are relatively high, ranging from 0.003 to 0.047, and the MAPE values are also elevated, with a peak of 21.1% at 5 cm. In the Up-direction, the Linear model performs even worse, with R^2^ values of −156.7%, −30.1%, and −122.7%, suggesting poor explanatory power. The RMSE values are high (0.417 to 0.522), and the MAPE values are excessively large, particularly at 30 cm (201.2%), further indicating poor prediction accuracy and instability. Overall, the Linear model struggles to provide reliable and accurate predictions across both directions.

**Figure 14:**
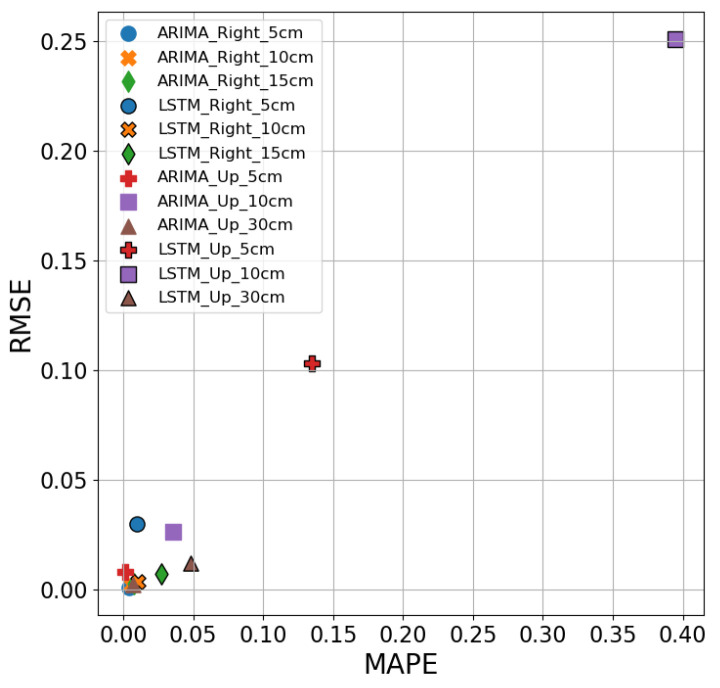
Comparison of MAPE and R^2^ of ARIMA and LSTM at different distances and directions.

Figure 14 compares the predictive performance of the ARIMA and LSTM models using MAPE and RMSE metrics. The ARIMA model (without marker edges) achieves superior accuracy and lower error rates across nearly all positions in both the Right and Up directions. For example, at 5 cm and 30 cm in the Up direction, ARIMA achieves MAPE values of 1.2% and 0.7%, respectively, with correspondingly low RMSE values, significantly outperforming the LSTM model. In contrast, the LSTM model (with marker edges) shows much higher MAPE values, such as 13.5% and 39.5% at the same positions, indicating a substantial prediction error. Moreover, LSTM yields negative R^2^ values across all Up-direction points, suggesting an inability to model upward ozone concentration trends. These findings highlight ARIMA’s advantage in linear, time-dependent data modeling, particularly in contexts with limited data points and strong autocorrelation.

**Figure 15:**
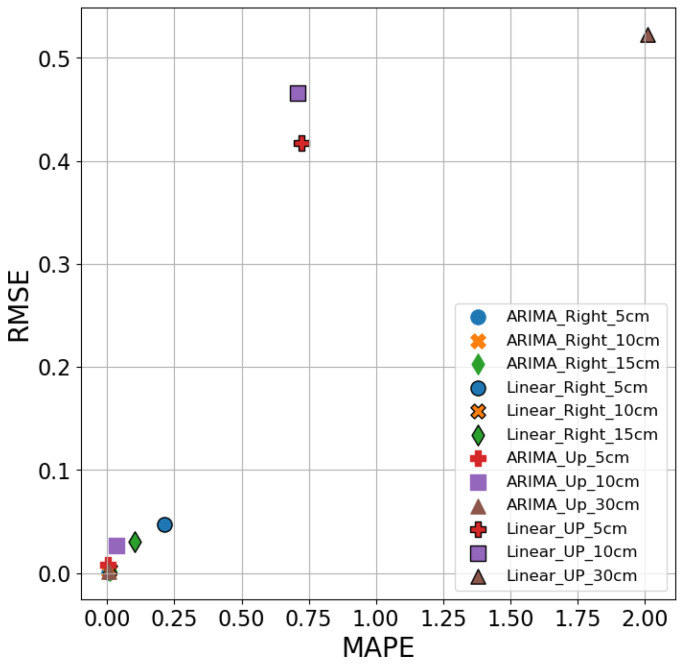
Comparison of RMSE and MAPE of ARIMA and LSTM at different distances and directions.

Figure 15 compares the predictive performance of the ARIMA and Linear Regression models using MAPE and RMSE metrics. The ARIMA model (without marker edges) demonstrates consistent superiority across all distances and directions, with notably low MAPE and RMSE values. For instance, in the Right direction at 10 cm, ARIMA achieves a MAPE of 1.3%, whereas the Linear Regression model, while recording a low MAPE of 1.7%, likely suffers from overfitting due to limited data variability. More critically, at 5 cm and 15 cm in the Right direction, the Linear model’s MAPE increases to 11.38% and 11.35%, with high RMSE values, and performs even worse in the Up direction with MAPE values of 40.2%, 24.6%, and 7.5% at 5 cm, 10 cm, and 30 cm, respectively. The Linear model also exhibits extreme negative R^2^ values, such as −219.8% at 5 cm Right and −156.7% at 5 cm Up, indicating its inadequacy in capturing temporal trends. These comparisons affirm ARIMA’s robust performance in structured time series environments.

**Figure 16:**
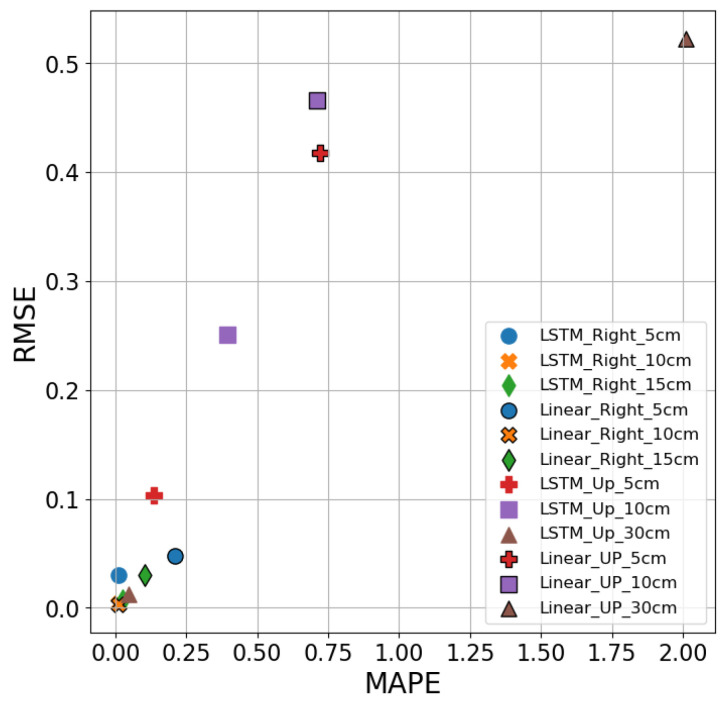
Comparison of RMSE and MAPE of LSTM model and linear regression model at different distances and directions.

Figure 16 compares the predictive performance of the LSTM and Linear Regression models using MAPE and RMSE metrics. Although both models underperform relative to ARIMA, the LSTM model (without marker edges) generally offers more consistent predictive accuracy than the Linear model, particularly in the Right direction. For instance, in the Right direction, the Linear Regression model records MAPE values above 11% at both 5 cm and 15 cm, while LSTM maintains lower MAPE and RMSE values in these positions. However, both models show poor performance in the Up direction, with LSTM’s MAPE reaching 39.5% at 10 cm and Linear Regression reaching 40.2% at 5 cm. The Linear model further suffers from erratic RMSE values and severely negative R^2^ across most locations, while LSTM, though still prone to errors in nonlinear environments, maintains slightly better adaptability. Overall, LSTM surpasses Linear Regression in handling time series predictions, though it still lags behind ARIMA in terms of precision and reliability.

The ARIMA model demonstrates superior prediction accuracy, especially at the Up-direction. For instance, at 10 cm and 30 cm Up, the MAPE values for the ARIMA model are 3.6% and 0.7%, compared to 39.5% and 4.8% for the LSTM model, indicating that ARIMA produces smaller prediction errors. Notably, at 5 cm Up, the ARIMA model’s MAPE is only 1.24%, while the LSTM model’s MAPE is 13.5%, showing a significant error difference. In contrast, the Linear Regression model performs poorly across all Up-direction measurements, with MAPE values reaching 72.1%, 70.9%, and 201.2% at 5 cm, 10 cm, and 30 cm, respectively—highlighting its inability to generalize effectively in more variable conditions. Similarly, in terms of R^2^, Linear Regression consistently yields negative values, confirming its lack of explanatory power in this context. Overall, ARIMA exhibits higher accuracy and stability across different positions, outperforming both LSTM and Linear Regression, which show larger errors in most cases. This superior performance can be attributed to ARIMA’s linear nature, which is well-suited for capturing linear relationships in stable, short-span data. In contrast, LSTM, which excels in modeling long-term dependencies and nonlinear patterns, underperforms in simpler linear contexts, while Linear Regression struggles due to its overly simplistic assumptions.

Data from the Right side of the measurement positions performs better than the Up side, likely due to stronger time correlations that allow ARIMA to more effectively utilize the data’s temporal dynamics. Additionally, data closer to the light source yields better predictions, possibly because of its higher signal reliability, making it easier for the model to learn patterns and relationships during training. The stability and continuity of these data sets further enhance the model’s predictive accuracy.

In this study, we applied the ARIMA model to predict indoor ozone concentrations for environmental safety evaluation. Actual data were collected in collaboration with the National Synchrotron Radiation Research Center (NSRRC). The modeling process included key steps such as data preprocessing (integration and differencing), stationarity testing, parameter tuning, and model diagnostics. Notably, the Minimum Description Length (MDL) principle was integrated into the model selection process to objectively balance model complexity and goodness-of-fit, thus preventing overfitting and improving generalizability. Key findings include the importance of careful data selection to avoid spurious trends, the accurate estimation of model parameters using tools like ACF, PACF, AIC, BIC, and MDL, and the validation of model adequacy through residual analysis and white noise tests. Comprehensive evaluation using RMSE, MAPE, and R^2^ metrics confirmed the ARIMA model’s superior performance compared to both LSTM and Linear Regression, especially when data are limited or highly structured in time.

The model’s consistent and interpretable performance underscores its potential as a robust tool for real-time environmental monitoring and prediction. Future work will aim to refine the ARIMA framework and expand its application to other pollutants and environmental indicators, contributing to more effective environmental decision-making.

## 5. Conclusions

This study presented a sensor-based ozone monitoring and prediction framework for laboratory environments where ozone may be generated during experimental operations. Ozone concentration data were collected using a UV absorption ozone analyzer installed at the National Synchrotron Radiation Research Center (NSRRC) and analyzed as a time-series dataset. Three prediction models—ARIMA, LSTM, and linear regression—were evaluated for short-term ozone concentration forecasting.

Experimental results demonstrate that the ARIMA model consistently provides the most reliable prediction performance for the small-sample dataset investigated in this study. In particular, ARIMA achieved the highest predictive accuracy across multiple measurement locations, with the best performance observed at 30 cm in the Up direction (R^2^ = 79.7%, MAPE = 0.68%). These results indicate that classical time-series models can effectively capture temporal patterns in sensor-generated environmental data, especially in environments where data availability is limited.

The proposed approach highlights the importance of combining sensing devices with predictive data analysis techniques to enhance environmental monitoring capabilities. Such sensor-enabled predictive systems can support real-time safety monitoring, early warning mechanisms, and ventilation control strategies in laboratory facilities where ozone generation may occur.

Future work will focus on extending the proposed framework to more comprehensive sensing scenarios. In particular, future experiments will investigate ozone concentration dynamics under different synchrotron operating conditions, including variations in beam energy and beam current levels, which may influence ozone generation rates and spatial distribution. Incorporating measurements obtained under multiple operational conditions would enable the development of more generalized prediction models for ozone concentration behavior in synchrotron facilities. Additionally, future studies will explore the integration of the proposed prediction model with real-time sensor monitoring systems, allowing continuous ozone sensing data to be processed for predictive safety alerts and ventilation control strategies. Such developments could further enhance sensor-based environmental safety management in laboratory environments where ozone generation may occur.

## Figures and Tables

**Table 1:** Model 106-L ozone monitor specifications.

Measurement Range	0–100 ppm
Limit of Detection (10 s average, 2σ)	0.02 ppm
Resolution	0.01 ppm
Sensitivity Drift	<1%/day, <3%/year
Accuracy	Greater of 0.01 ppm or 2% of reading
Measurement Time and Frequency	2 s, 0.5 Hz
Data Averaging Options	10 s, 1 min, 5 min, and 1 h
Ozone Units	ppb, ppm
Operating Temperature Range	0 to 50 °C
Pressure Units	mbar, torr
T and P Corrected	Yes
DewLine™ for Humidity Control	Yes
Calibration Procedures	NIST Traceable; Annual calibration recommended

**Table 2:** Measurement conditions.

Experiment	Direction	Distance
1	Right side	5 cm
2	Right side	10 cm
3	Right side	15 cm
4	Up side	5 cm
5	Up side	10 cm
6	Up side	30 cm

**Table 3:** The right side shows the results for ARIMA and LSTM models.

Experiment Condition	Direction Right	Model	Performance Indicator		
			R^2^	RMSE	MAPE
1	5 cm	ARIMA	89.5%	0.001	0.41%
		Linear	−22K%	0.047	21.1%
		LSTM	73.2%	0.030	1.00%
2	10 cm	ARIMA	86.3%	0.002	0.55%
		Linear	66.4%	0.003	1.04%
		LSTM	35.5%	0.004	1.34%
3	15 cm	ARIMA	81.1%	0.003	0.79%
		Linear	−2.5K%	0.030	10.3%
		LSTM	−1.2%	0.007	2.70%

**Table 4:** The up side shows the results for ARIMA and LSTM models.

Experiment Condition	Direction Up	Model	Performance Indicator		
			R^2^	RMSE	MAPE
4	5 cm	ARIMA	32.7%	0.008	1.24%
		Linear	−157K%	0.418	72.1%
		LSTM	−46.5%	0.103	13.5%
5	10 cm	ARIMA	18.0%	0.027	3.59%
		Linear	−30K%	0.466	70.9%
		LSTM	−86.3%	0.251	39.5%
6	30 cm	ARIMA	79.7%	0.002	0.68%
		Linear	−1.23M%	0.522	201.2%
		LSTM	−7.1%	0.012	4.8%

## Data Availability

The original contributions presented in this study are included in the article. Further inquiries can be directed to the corresponding author.

## Abbreviations

The following abbreviations are used in this manuscript:

ADF	Augmented Dickey–Fuller
AIC	Akaike Information Criterion
ARIMA	Autoregressive Integrated Moving Average
BIC	Bayesian Information Criterion
LSTM	Long Short-Term Memory
MAPE	Mean Absolute Percentage Error
MDL	Minimum Description Length
NSRRC	National Synchrotron Radiation Research Center
PACF	Partial Autocorrelation Function
RMSE	Root Mean Square Error
